# Improving the Safety of ADHD Medication Prescribing in Patients with Psychosis: A Quality Improvement Project in an “Early Intervention in Psychosis” Service

**DOI:** 10.1192/bjo.2026.11265

**Published:** 2026-06-30

**Authors:** Omolade Dada, Mosunmola Aire

**Affiliations:** Kent and Medway Mental Health Trust, Gillingham, United Kingdom

## Abstract

**Aims:**

Attention Deficit Hyperactivity Disorder (ADHD) commonly co-occurs with psychotic disorders. First-line treatment for ADHD involves stimulant medications, which increase dopaminergic transmission and may exacerbate psychotic symptoms in vulnerable individuals. Patients within Early Intervention in Psychosis (EIP) services who are prescribed stimulant medication therefore represent a potential prescribing safety risk, particularly when antipsychotics are co-prescribed.

Aims were to assess documentation of stimulant-associated psychosis risk and monitoring practices in patients with comorbid ADHD and psychosis within an EIP service, and to identify areas for quality improvement.

**Methods:**

A retrospective cross-sectional review of electronic patient records was conducted for all patients under the North Kent EIP service. Patients with at least one episode of psychosis and a formal diagnosis of ADHD were identified. Data collected included ADHD and psychosis diagnoses, prescribed stimulant and antipsychotic medications, documentation of stimulant-related psychosis risk within risk assessments, evidence of monitoring plans, and records of stimulant discontinuation or medication review due to psychotic symptoms.

**Results:**

Of 152 patients under the EIP caseload, 16 (10.5%) had a confirmed diagnosis of ADHD. Four patients were prescribed stimulant medication, two of whom were concurrently prescribed antipsychotic medication. No risk assessments documented the potential for stimulant-associated psychosis exacerbation, and none included a specific monitoring plan addressing this risk. ADHD was referenced as a comorbidity in 75% of risk assessments. No cases of documented stimulant discontinuation due to psychosis were identified.

**Conclusion:**

Although ADHD was frequently recognised as a relevant comorbidity, risk assessments did not address the potential for stimulant-associated psychosis exacerbation or include monitoring plans. This represents a gap in prescribing safety within EIP services. A targeted educational intervention has been developed to improve staff awareness and documentation so that improvements can be made in the safety of prescribing amongst this underrepresented group.

